# Two new species of *Tovlinius* Zaitzev, from China, with a key to the genera of Bombyliinae from China and a second key to the world species (Diptera, Bombyliidae, Bombyliinae, Bombyliini)

**DOI:** 10.3897/zookeys.153.2031

**Published:** 2011-12-09

**Authors:** Gang Yao, Ding Yang, Neal L. Evenhuis

**Affiliations:** 1 Department of Entomology, China Agricultural University, Beijing 100193, China; 2 Zhejiang Forestry Academy, 399 Liuhe Street, Hangzhou, Zhejiang 310020, China; 3 Department of Natural Science, Bishop Museum, 1525 Bernice Street, Honolulu, Hawaii 96817-2704, USA

**Keywords:** Diptera, Bombyliidae, *Tovlinius*, new species, China, Palaearctic, taxonomy

## Abstract

The genus *Tovlinius* Zaitzev is a Palaearctic genus with just one previously described species, *Tovlinius albissimus* Zaitzev. *Tovlinius* is here recorded from China for the first time, and two new species *Tovlinius pyramidatus*  **sp. n.** and *Tovlinius turriformis* **sp. n.** are described and illustrated. A key to the genera of Bombyliinae from China and a second key to the World species of *Tovlinius* are also presented herein.

## Introduction

The genus *Tovlinius* Zaitzev, 1979 belongs to the tribe Bombyliini of Bombyliinae (Evenhuis and Greathead 1999). Species of this genus are easily identified by the following characters: Proboscis 3.5 times longer than head; eyes of males not converging in the front of ocellar triangle; scape not swollen, tip of first flagellomere with clear terminal stylus; mid-tibial spur absent; vein R_1_-R_2+3_ absent (2 submarginal cells); wing with basicostal infuscation tending to be more intense along margins of veins; wing distinctly swollen close to tip, cell r_5_ closed and with long stalk; abdomen broad; body sometimes with pale markings (Greathead and Evenhuis 1997; Zaitzev 1979). *Tovlinius* previously included just one known species (*Tovlinius albissimus* Zaitzev) which is known only from Kazakhstan (Evenhuis and Greathead 1999). In the present paper, two new species, *Tovlinius pyramidatus* sp. n. and *Tovlinius turriformis* sp. n., are added, both from Sichuan Province in western China. A key to the genera of the Bombyliinae from China and a key to World species of the genus *Tovlinius* are presented.

## Material and methods

Specimens were studied and illustrated with a ZEISS Stemi 2000-c stereomicroscope. Genitalic preparations were made by macerating the apical portion of the abdomen in cold 10% NaOH for 12–15 hours. After examination they were transferred to fresh glycerin and stored in a microvial pinned below the specimen. Photographs of the wing and adult abdomen were taken with a digital camera (Canon 450D) and modified with Adobe Photoshop. Voucher specimens examined are deposited in the Nankai University, Tianjin (NKU) and the Shanghai Entomological Museum, Chinese Academy of Sciences, Shanghai (SEMCAS). The following abbreviations are used: *ad* – anterodorsal, *av* – anteroventral, *pd* – posterodorsal, *pv* – posteroventral, dm – discal cell.

### Key to genera of Bombyliinae from China

**Table:** 

1	Wing with discal cell broadest towards apex, vein M-M meeting M-CuA at an obtuse angle; radial sector broad with costa tending to bulge forwards near apex of wing, wing broadest towards apex	2
–	Wing with discal cell broadest near middle, vein M-M meeting M-CuA at not much more than a right angle; radial sector not enlarged with costa more or less straight along fore margin of wing, wing not broader toward apex	3
2	Antennal scape greatly swollen; mid-tibial spur present; vein R_1_-R_2+3_ present in some species (2 or 3 submarginal cells); abdomen elongate ovate; body hairs long	*Conophorus* Becker
–	Antennal scape at most only moderately swollen; mid-tibial spur absent; vein R_1_-R_2+3 _absent (2 submarginal cells); abdomen elongate ovate or short and broad; body hairs short	*Tovlinius* Zaitzev
3	Head usually as broad as thorax; hind margin of eyes indented; wings often small, narrowed at base with at least alula reduced; body usually more elongate, even narrow conical or cylindrical	*Anastoechus* Osten Sacken
–	Head usually distinctly narrower than thorax; eyes not indented on hind margin; wing usually large, broad at base with anal lobe, alula and squama well developed; body always broad	4
4	Wing with cell br as long as cell bm; vein R-M shorter than vein M-M, occasionally almost equal in length; vestiture fine and silky with a clipped appearance at least on occiput and thorax, hairs white to straw-yellow or brown shading to paler on underside	*Systoechus* Loew
–	Wing with cell br longer than cell bm; vein R-M as long as vein M-M; vestiture various, often with black hairs and scale patches; wing pattern various, sometimes with a clear-cut dark infuscation and with isolated dark spots	5
5	Body length usually less than10 mm, wing entirely infuscated	*Euchariomyia* Bigot
–	Body length usually longer than 10 mm, wing at least with hyaline spots	6
6	Antennal scape about twice length of pedicel; upper facets of eyes of males not enlarged; face short and with short sparse hairs only; wing base black, remainder hyaline or tinged yellowish; body hair short and with a clipped appearance, usually with abundant black elements contrasting with areas of white, orange or grey	*Bombomyia* Greathead
–	Antennal scape more than twice length of pedicel; upper facets of eyes of males enlarged; face long, prominent and with long hairs; wing pattern, if present, confined to basicostal area, often diffuse and sometimes with isolated spots, wing rarely completely infuscated; body hair usually long, sometimes tufted and rarely with a clipped appearance, usually predominantly white to yellow or brown with a few black elements, if predominantly black then without a contrasting pattern of pale hair	*Bombylius* Linnaeus

### Key to world species of *Tovlinius*

**Table:** 

1	Antennal scape covered with long dense black and white hairs; legs mostly covered with yellow hairs and bristles	2
–	Antennal scape covered with long white hairs; legs mostly covered with white scales and black bristles	*Tovlinius albissimus* Zaitzev
2	Antenna black, first flagellomere bare; legs black except tibiae yellow; haltere dark brown; epandrium slightly narrowing toward tip in dorsal view	*Tovlinius pyramidatus* sp. n.
–	Antenna black except joints brown, first flagellomere with sparse white scales; Legs yellow except femora black; haltere black; epandrium almost parallel-sided in dorsal view	*Tovlinius turriformis* sp. n.


#### Tovlinius pyramidatus sp. n.

urn:lsid:zoobank.org:act:2E882E27-F45A-4C55-BE2E-0FE0310BB51E

http://species-id.net/wiki/Tovlinius_pyramidatus

Figs 1–7 

##### Diagnosis.

Antenna black, first flagellomere elongate, bare. Scutellum with long dense white hairs. Wing uniformly weak brown except base brown; vein C with brush-like long black bristles, white hairs, and yellowish scales. Dorsum of abdomen with long dense white erect hairs and black bristles laterally becoming denser apically; legs black except tibiae yellow; haltere dark brown. Epandrium trapezoidal in lateral view, slightly narrowing toward tip in dorsal view; epiphallus pyramid-shaped.

##### Description.

Male. Body length 10 mm, wing length 9 mm.

Head black. Hairs on head black and white; frons narrowing distally, with long dense black erect hairs; face with long dense white erect hairs; occiput with dense white erect hairs and long sparse black hairs. Antenna black; scape elongate with long dense black and white hairs; first flagellomere elongate, bare, with stylus at tip. Proboscis broken.

**Figures 1–7.**
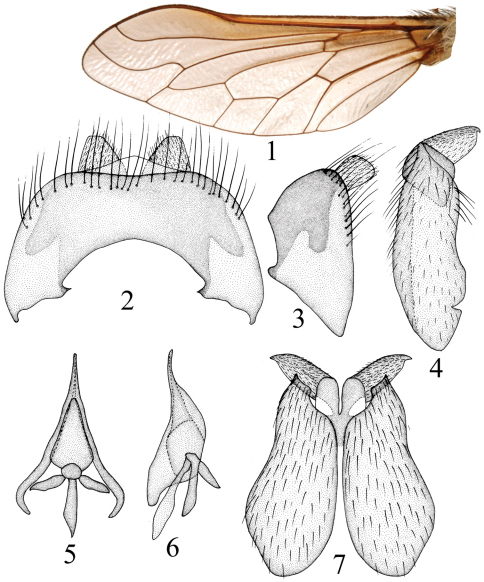
*Tovlinius pyramidatus* sp. n. wing and male genitalia **1** wing, dorsal view **2** epandrium and cercus, dorsal view **3** epandrium and cercus, lateral view **4** gonocoxite and gonostylus, lateral view **5** phallus, dorsal view **6** phallus, lateral view **7** gonocoxite and gonostyli, ventral view.

Thorax black. Hairs on thorax mostly white; postpronotal lobe with long dense white hairs, mesonotum with long dense white hairs; thorax with sparse white hairs on anepisternum, and with long dense white hairs on katepisternum. Scutellum black with long dense white hairs. Legs black except tibiae yellow. Hairs on legs mostly yellow, bristles yellow. Femora with dense white hairs and scales; tibiae and tarsi with short yellow hairs and white scales. Hind femur with three *av*. Fore tibia with seven *ad*, eight *pd*, six *pv*; mid tibia with seven *ad*, eight *pd*, eight *av*, seven *pv*; hind tibia with eight *ad*, seven *pd*, six *av*, six *pv*. Wing (Fig. 1) uniformly weak brown except base brown. Vein r-m close to tip of cell dm, cell r_5_ closed. Base of vein C with brush-like long black bristles, white hairs, and yellowish scales. Haltere dark brown.

Abdomen black. Hairs on abdomen mostly white; with long dense white erect hairs and sparse yellowish hairs dorsally, and with black bristles laterally that become denser apically. Sternites black except posterior edge and middle brown, sternites with long dense white erect and recumbent hairs.

Male genitalia (Figs 2–7). Epandrium trapezoidal in lateral view, distinctly higher than long, distinctly wider than long in dorsal view; cercus well exposed in lateral view; gonocoxite distinctly narrowing apically in ventral view; gonostylus oval with apex pointed (seen laterally); epiphallus pyramid-shaped with apex very narrow , epiphallus with narrow, long, and curved tip in lateral view.

Female. Unknown.

##### Type material.

Holotype male, CHINA: Sichuan, Hongyuanxian, Shuajingsi (32°00'52"N, 102°36'59"E), 5.VIII.1983, Leyi Zheng (NKU).

##### Distribution.

China (Sichuan).

##### Etymology.

The species is named after the pyramid-form of the epiphallus.

##### Remarks.

*Tovlinius pyramidatus* is similar to *Tovlinius albissimus* Zaitzev, but it can be differentiated from the latter by the following points: Scape covered with long dense black and white hairs; legs black except tibiae yellow, mostly covered with yellow hairs and bristles; haltere dark brown; epandrium slightly narrowing toward tip in dorsal view. In *Tovlinius albissimus*, the scape is covered with the long white hairs; the legs are yellow and covered with the white scales; the haltere is pale yellow; the epandrium is distinctly narrowing toward the tip in dorsal view (Zaitzev 1979).

#### Tovlinius turriformis sp. n.

urn:lsid:zoobank.org:act:EEDE376A-9368-4247-9403-38A795B35FEB

http://species-id.net/wiki/Tovlinius_turriformis

Figs 8–14 

##### Diagnosis.

Antenna black except joints brown, first flagellomere elongate with sparse white scales. Scutellum with long white hairs, posterior edge with white bristles. Wing uniformly weak brown; base of vein C with brush-like long black bristles, white hairs, and white scales. Dorsum of abdomen with long dense white erect hairs and white bristles laterally, hairs and bristles becoming denser apically; legs yellow except femora black; haltere black. Epandrium trapezoidal in lateral view, almost parallel-sided in dorsal view; epiphallus turriform in dorsal view.

##### Description.

Male. Body length 10 mm, wing length 9 mm.

Head black. Hairs on head black and white; frons narrowing distally, with long dense black and white erect hairs; face with long dense white erect hairs; occiput with long dense white erect hairs and long sparse black hairs. Antenna black except joints brown; scape elongate with long dense black and white scales; first flagellomere elongate with sparse white scales, and tip with a stylus. Proboscis black, nearly five times longer than head.

**Figures 8–14.**
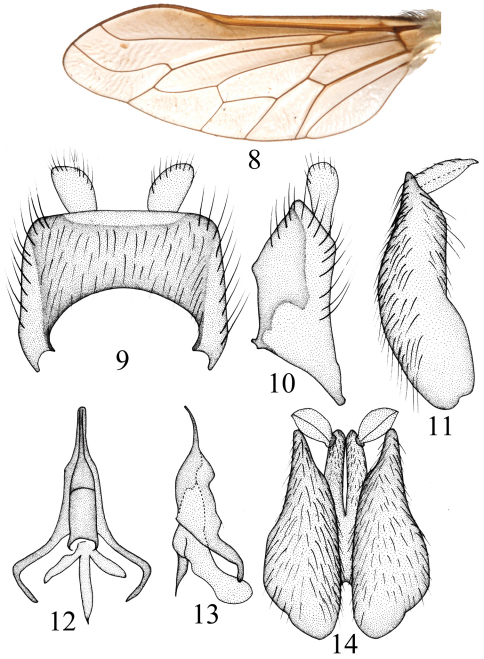
*Tovlinius turriformis* sp. n. wing and male genitalia **8** wing, dorsal view **9** epandrium and cercus, dorsal view **10** epandrium and cercus, lateral view **11** gonocoxite and gonostylus, lateral view **12** phallus, dorsal view **13** phallus, lateral view **14** gonocoxite and gonostyli, ventral view.

Thorax black with brown pollen. Hairs on thorax mostly white; postpronotal lobe with long dense white hairs, mesonotum with sparse long white hairs; anepisternum and katepisternum with long dense white hairs. Scutellum black with long white hairs, posterior edge with white bristles. Legs yellow except femora black. Hairs on legs mostly yellow, bristles yellow, scales white. Femora with dense white hairs and scales; tibiae and tarsi with short yellow hairs and white scales. Hind femur with three *ad*, three *av*, and three *pv*. Fore tibia with seven *ad*, eight *pd*, five *av*, and six *pv*; mid tibia with seven *ad*, seven *pd*, eight *av*, and six *pv*; hind tibia with eight *ad*, seven *pd*, seven *av*, and six *pv*. Wing (Fig. 8) uniformly weak brown. Vein r-m close to tip of cell dm, cell r_5_ closed. Base of vein C with brush-like long black bristles, white hairs, and white scales. Haltere black.

Abdomen black. Hairs on abdomen mostly white; dorsum with long dense white erect hairs and lateral surface with white bristles that become denser apically, tergites 4-7 with some black bristles laterally. Sternites with long dense white hairs.

Male genitalia (Figs 9–14). Epandrium trapezoidal, distinctly higher than long, slightly wider than long in dorsal view; cercus well exposed in lateral view; gonocoxite distinctly narrowing apically in ventral view; gonostylus oval, its tip acute in lateral view; epiphallus turriform, obtuse at tip in dorsal view, epiphallus with a narrow, long, and curved tip in lateral view.

Female. Unknown.

##### Type material.

Holotype male, CHINA: Sichuan, Maerkang (31°54'21"N, 102°12'23"E), 30.VII.1986, Tianqi Wang (SEMCAS).

##### Distribution.

China (Sichuan).

**Etymology.** The species epithet derives from the Latin “*turri* [=tower, turret] + *formis*” [= form]; referring to the tower-like shape of the epiphallus.

##### Remarks.

*Tovlinius turriformis* is similar to *Tovlinius albissimus* Zaitzev, but it can be separated from the latter by the following points: Antenna black except joints brown; legs yellow except femora black, mostly covered with yellow hairs and bristles, and white scales; haltere black; epandrium almost parallel-sided in dorsal view. In *Tovlinius albissimus*, the basal two antennal segments are yellow; the legs are yellow and covered with the white scales; the halteres are pale yellow; the epandrium is distinctly narrowing toward the tip in dorsal view (Zaitzev 1979).

## Supplementary Material

XML Treatment for Tovlinius pyramidatus

XML Treatment for Tovlinius turriformis
